# The emerging role of IL-17A across different types of radiation-induced normal tissue injuries

**DOI:** 10.1177/17588359251389745

**Published:** 2025-11-09

**Authors:** Ziqi Wang, Jann-Birger Laugsand, Guanglin Cui

**Affiliations:** Medical Radiography and Imaging, Mudanjiang Medical University, Mudanjiang, China; Faculty of Health Science, Nord University, Campus Levanger, Levanger, Norway; Faculty of Health Science, Nord University, Høgskolevegen 27, Campus Levanger, Levanger 7600, Norway

**Keywords:** immunopathogenesis, interleukin-17, radiation-induced tissue injury, radiotherapy, therapy

## Abstract

Radiation-induced normal tissue injuries (RITIs) are the main complications that can significantly limit the use of radiotherapy and affect clinical outcomes in patients with cancer. This article aims to review current literature on the role of proinflammatory cytokine interleukin (IL)-17A in different types of RITIs. While irradiation significantly increases IL-17A expression in normal tissues, activated IL-17A plays a dual role of both promoting and protecting against RITIs, depending on the tissue type. These novel findings have led to a strong interest in evaluating the therapeutic potential of targeting IL-17A/IL-17 receptor signals in RITIs in different normal tissues. Preliminary results from preclinical animal models have shown that blocking IL-17A/IL-17 receptor signaling after irradiation significantly reduces the pathogenesis of RITIs in the skin and lung but enhances it in the intestine and oral mucosa. However, the mechanisms underlying the tissue-dependent dual roles of IL-17A in RITIs remain elusive. Therefore, future studies focusing on the precise role and mechanism of IL-17A action in different RITIs are needed.

## Background

The efficacy of radiotherapy (RT) in treating human cancers has been widely recognized. However, radiation-induced normal tissue injuries (RITIs) remain one of the major clinical concerns in patients with cancer undergoing RT.^1–7^ Clinically, RITIs generally refer to the damage to healthy tissues that seriously restricts the therapeutic effectiveness of RT, leading to poor prognosis.^2,8^ Therefore, identifying the factors contributing to the development of RITIs is critically important. Mechanistic studies^9–13^ have identified that multiple factors, for example, free radicals and reactive oxygen species (ROS) and subsequent DNA breakages, play an essential role in sustained and uncontrolled inflammation in the surrounding normal tissues/organs caused by RT and lead to RITIs. Moreover, these substances can induce the activation of inflammatory cytokines, which in turn lead to the subsequent activation of different transcription factors to trigger inflammatory cascades to enhance tissue inflammation and injury.^14–24^ Early studies have highlighted changes in the profiles of inflammatory cytokines during the pathophysiological process of RITIs and their significance. Cytokines, such as interleukin (IL)-1β, IL-6, IL-33, and tumor necrosis factor (TNF)-α, play central roles in the induction of radiation-induced tissue inflammation and injury.^17,25–29^ IL-17A is a key member and the most studied and best-described subtype of the IL-17 cytokine family, which comprises a group of signaling proteins including IL-17A, IL-17B, IL-17C, IL-17D, IL-17E (also called IL-25), and IL-17F.^30^ IL-17 receptor family consists of five subunits, including IL-17 RA, IL-17RB, IL-17RC, IL-17RD, and IL-17RE, that are broadly *expressed* in different tissues and cells and critically mediate the biological action of IL-17 cytokines.^30^ A large body of research highlights the proinflammatory role of IL-17A in the regulation of host immune defense, especially in response to bacterial, mycobacterial, and fungal pathogens.^30,31^ In addition, highly activated IL-17A signaling plays a critical role in the initiation of inflammation in different tissues and is associated with the development of autoimmune and inflammatory diseases.^30^ IL-17A is also implicated in some cancers, such as breast, gastric, and colorectal cancers, for promoting the development, progression, and metastasis through immune suppression, increased angiogenesis, and stimulation of cell proliferation.^30,32^ IL-17RA and IL-17RC form a complex structure and function as the main functional receptors for IL-17A,^30^ different agents or approaches, such as anti-IL-17RA or IL-17RC monoclonal antibodies and genetic techniques, used to block the IL-17A signal, have shown therapeutic potential in the management of IL-17A-related autoimmune and chronic inflammatory diseases and cancers.^33–36^

In addition to its role in the initiation and maintenance of tissue inflammation and the development of inflammatory and autoimmune diseases and cancers, IL-17A has also been shown to be involved in the development of radiation-induced tissue inflammation and injury.^37–40^ Numerous investigations have provided compelling evidence that ionizing radiation leads to the increased accumulation of IL-17A-expressing cells and elevated expression levels of IL-17A in surrounding normal tissues, which are closely associated with the acute toxicity of radiation^27,41^ and RITIs.^14,16,40,42^ Animal studies have shown that post-irradiation, while activated IL-17A signaling leads to injuries in the lung and skin tissues,^14,40,43^ it has a protective effect of IL-17A against tissue injury in the intestine and oral mucosa.^44,45^

Given the increasing number of studies investigating the emerging role of IL-17A in radiation-induced inflammation and RITIs, this review highlights recent findings and provides an understanding of the distinct functions and mechanisms of IL-17A in different RITIs. In addition, the translational significance of targeting IL-17A/IL-17 receptor signals as a novel pharmacotherapeutic option for the management of RITIs is proposed and discussed.

## RT-induced activation of IL-17A

Cytokines are key mediators of inflammation. Hence, it is not surprising that ionizing radiation, one of the “dangerous” triggers of inflammation, leads to an increased expression of inflammatory cytokines,^17,26,27,46,47^ by activating the transcription factor nuclear factor-kappa B (NF-κB) signaling pathway.^48^

The critical role of IL-17A in the initiation and maintenance of tissue inflammation is well known.^30^ Published data have documented a significant increase in the populations of Th17 or IL-17A-positive cells and expression levels of IL-17A in various irradiated tissues.^39,40,43^ Mieczkowska et al.^40^ demonstrated a positive correlation between the increased IL-17A level and the severity of tissue injury in irradiated skin, providing evidence for the contribution of IL-17A to the disease progression of radiation-induced dermatitis (RID) in mice. Furthermore, studies have shown elevated IL-17A expression levels and IL-17A-positive cell density in normal lung tissues after exposure to irradiation.^14,49–52^ Studies also reported that changes in IL-17A-positive cell populations can predict the therapeutic response of RT in cancers.^43,44,53–55^ For example, Theobald et al.^56^ revealed that increased numbers of Th17 cells in blood were associated with resistance and recurrence in cervical cancer patients after chemoradiotherapy, suggesting their potential as predictors of therapeutic response. Therefore, increasing evidence suggests the involvement of the activated IL-17A signal in RITIs.

## Effects of IL-17A on the pathogenesis of different RITIs

### IL-17A promotes RITIs in the esophageal, skin, and lung tissues

#### Radiation-induced esophageal injury

S100A8/A9 is a member of the calcium-binding S100 protein family produced by immune cells. It is not expressed in healthy tissues, but it is significantly activated in inflammatory conditions, such as inflammatory diseases and cancers.^57,58^ Thus, S100A8/A9 is a potential biomarker of tissue inflammation and activation of proinflammatory cytokines.^59^ Lee et al.^60^ showed that X-ray irradiation markedly induces S100A8 expression in the hyperplastic epidermis of mice. In the context of radiation-induced esophageal injury (RIEI), Yao et al.^61^ showed a marked increase in expressions of S100A8/A9 in the irradiated rat esophagus, accompanied by activation of the IL-17A signaling pathway and a strong inflammatory response. Furthermore, they found that the activated IL-17A signaling pathway was associated with the development of RIEI in rats.^61^ However, the exact significance of S100A8/A9 in predicting IL-17A activation in RIEI has not been clearly established. Further studies are required to verify these findings.

#### Radiation-induced dermatitis

Previous studies have reported an increase in the expression of proinflammatory cytokines, such as IL-1, IL-6, IL-8, TNF-α, and transforming growth factor (TGF)-β, in irradiated skin tissues, leading to an inflammatory response.^22^ Recently, Liao et al.^43^ reported an elevation in the numbers of IL-17-expressing T-cells, rather than Th17 cells, in the skin of C57BL/6J wild-type (WT) mice exposed to a single X-ray dose of 25 Gy. In C57BL/6J Tcrd^−/−^ mice (which are deficient in γδ-T cells), RID was greatly reduced,^43^ supporting the importance of IL-17-expressing γδ-T cells in the immunopathogenesis of RID. Furthermore, Mieczkowska et al.^40^ have recently reported a significant upregulation of IL-17A and inflammatory marker S100A8/A9 in the skin tissue in response to radiation, which was positively correlated with the severity of RID in mice. Malecka et al.^62^ have reported that stromal fibroblasts modulate the effect of dendritic cells (DCs) on the maintenance of the IL-23/IL-17A immune response to ionizing radiation stimulation. Interestingly, data analysis by Mieczkowska et al.^40^ showed a positive association between the elevated IL-17A expression and increased DC populations in irradiated skin,^40^ suggesting that both stromal fibroblasts and DCs are involved in the modulation of IL-17A activation in response to ionizing irradiation.^63,64^ In line with these findings, animal studies demonstrated that inhibition of IL-17A signaling by an IL-17A antibody prevented severe RID in mice,^40^ supporting the role of radiation-induced IL-17A activation in the development of RID.

#### Radiation-induced pulmonary injury

Regarding the involvement of IL-17A in the development of radiation-induced pulmonary injury (RIPI), both in animal models and in patients with cancer undergoing RT, has been preliminarily studied and provided supportive evidence. For example, several studies have revealed an elevated expression level and/or population of IL-17A-positive cells in lung tissues after exposure to irradiation.^14,49–52^ Wang et al.^65^ have shown that both the population of IL-17A-positive cells in lung tissues and the expression level of IL-17A in bronchoalveolar lavage fluid started to increase at 1 week, peaked at 4 weeks, and subsequently started to decline at 8 weeks after irradiation. An administration of dexamethasone significantly reduced the level of IL-17A and attenuated the severity of RIPI by reducing the density of inflammatory cells infiltrating into the lung tissues.^65^ Furthermore, they showed that the inhibitory effect of blocking the high mobility group box-1 (HMGB1) protein (an immune molecule implicated in the pathogenesis of lung diseases^66^) on the development of RIPI occurred through a decreased expression level of IL-17A in mouse models.^67^ Paun et al.^14^ further found that abundant IL-17A-expressing Th17 cells were observed in the pulmonary tissue and were associated with the development of pneumonitis with fibrosis after exposure to irradiation. However, IL-17A deficiency in mice significantly reduced the occurrence of both fibrosis and pneumonitis after exposure to radiation, confirming that IL-17A is deeply involved in the development of RIPI.^14^ Wang et al.^68^ provided more evidence to support the importance of IL-17A in the development of RIPI and showed that blocking IL-17A signaling by IL-17A-neutralizing antibodies significantly reduced the occurrence (percentage) of severe alveolitis (grades II and III) induced by single-dose irradiation of the thorax in mice. Moreover, Liu et al.^38^ found that activation of IL-17A by irradiation suppressed autophagy in human lung epithelial cells (BEAS-2B) and enhanced the process of RIPL. Recently, Liu et al.^69^ examined the impact of an anti-programmed cell death protein 1 (anti-PD-1) monoclonal antibody (an immune checkpoint inhibitor) combined with RT on the development of lung injury in PD-1-deficient (PD-1^−/−^ KO) mice. They reported that PD-1^−/−^ KO mice exhibited a high rate of RIPI in bilateral lungs after irradiation, which was associated with increased IL-17A expression. The administration of anti-IL-17A antibody decreased the expression levels of other RIPI-related factors, interferon-γ, TNF-α, IL-6, RAR-related orphan receptor, and TGF-β1 and prolonged the survival of mice. Zhang et al.^70^ performed genome-wide microarray analysis in RIOI mice induced by whole thoracic irradiation and reported that upregulated differentially expressed genes were enriched in the p53 and IL-17A signaling pathways, underlining the importance of IL-17A in the process and progression of RIPI.

Regulatory T-cells (Tregs) are immunosuppressive cells that play a vital role in modulating tissue inflammation and repair.^71^ Tregs have been implicated in RIPI pathogenesis by dampening Th17 activation and decreasing IL-17A production, factors that aid the repair of damaged lung tissue.^72^ Thus, the accumulation and activation of Tregs in tissues lead to reduced Th17/IL-17A activation. However, the available literature on the roles of Tregs/IL-17A in pulmonary fibrosis remains somewhat contradictory.^73^ For example, Xiong et al.^74^ have reported that depletion of Tregs in mice by intraperitoneal injection with anti-CD25 monoclonal antibody (that targets Tregs and suppresses immune response) 2 h after 20 Gy 60CO γ-ray thoracic irradiation significantly delayed the development of radiation-induced pulmonary fibrosis by increasing infiltration of CD4^+^ Th and Th17 cells in lung tissues, which suggest a possible anti-fibrotic effect of Th17 cells on irradiation-induced pulmonary fibrosis. These conflicting results may reflect the complexity of the cellular sources of IL-17A. In addition, Th17 cells may convert into immunosuppressive FoxP3^+^ Treg cells or produce IL-10, which restricts IL-17A activity under appropriate conditions.^75^ Therefore, the precise mechanisms of Tregs/IL-17A action in RIPI development remain elusive.

Table 1 summarizes the studies that have reported the promotive effects of IL-17A on RITIs in the esophagus, skin, and lung tissues.

**Table 1.** Main studies on IL-17A are known as a promotive factor in the pathogenesis of RITIs.

Study (Ref.) and year	Study models		Main findings	Conclusions
	Animal	Human		
RIEI				
Yao et al.,^61^ 2022	Irradiated rat esophagus exposed to 0, 25, or 35 Gy irradiation. Bioinformatics analyses were performed		Activated IL-17 (marked by S100a8 and S100a9) signaling pathway and immune cell infiltration in RIEI	The activated IL-17A signaling pathway in rat RIEI
RID				
Liao et al.,^43^ 2017	C57BL/6J wild-type and γδ-T cell-deficient mice exposure to a single X-ray dose of 25 Gy		In response to irradiation, wild-type mice exhibited an increased population of IL-17A-expressing γδ-T cell in skin in wild-type mice. γδ-T cell-deficient mice showed a reduced occurrence of RID	IL-17A-expressing γδ-T cells as promoters for RID
Mieczkowska et al.,^40^ 2021	Mice treated with radiation at a single dose of 25 Gy. The level of IL-17A mRNA was quantified with real-time PCR		Upregulated expression level of IL-17A in irradiated skin was correlated with the severity of RID in mice	Upregulated IL-17A level was correlated with the severity of RID in mice
RIPI				
Cappuccini et al.,^51^ 2011	Mice exposure to thorax irradiation (a single dose of 15 Gy over their right hemithorax, using a linear accelerator (dose rate 4.7 Gy/min))		CD4^+^/CD25^+^/IL-17^+^ T-cells increased in the lung on day 21	Radiation induces an increased population of Th17 cells in the lung tissues
Brickey et al.,^50^ 2012	MyD88-deficient (Myd88^−/−^) mice treated with thoracic targeted γ-irradiation		Deficiency of MyD88 in mice exhibited a reduced RIPI, which was accompanied by elevated IL-17A level	Increased level of IL-17A in MyD88-induced protection against RIPI and lung fibrosis
Wang et al.,^68^ 2014	Irradiated mice receiving IL-17A-neutralizing antibody treatment, and the percentage of RIPI severity was observed		Administration of IL-17A-neutralizing antibody resulted in a reduced percentage of grade II and III alveolitis induced by irradiation in the treatment group (16%, *p* < 0.05) as compared with the radiation control group (72%) or placebo group (64%)	IL-17A was a promoter for the process of RIPI
Wang et al.,^65^ 2014	Mice are exposed to a single dose of 15 Gy		IL-17A started to increase at 1 week, peak at 4 weeks, and subsequently decline at 8 weeks after irradiation. Dexamethasone application reduced the level of IL-17A and attenuated the severity of RIPI by reducing the density of inflammatory cells infiltrated in the lung tissues. The protective effect of HMGB1 on RIPI was via the suppression of IL-17A expression in mice	The activated IL-17A signal during the process of RIPI
Trovo et al.,^52^ 2016		Cytokines were measured in 15 early-stage NSCLC patients who underwent to extreme hypofractionated regimen (52 Gy in 8 fractions) with SBRT, and 13 locally advanced NSCLC patients underwent to radical moderated hypofractionated regimen (60 Gy in 25 fractions) with IMRT	IL-17A plasma level was higher in NSCLC than in controls; on the first day after SBRT or IMRT, IL-17 plasma level was significantly increased as compared with before; however, it was reduced on the last day after SBRT	Increased IL-17A plasma level in SBRT and IMRT in NSCLC patients
Paun et al.,^14^ 2017	Irradiated IL-17^−/−^, Tlr2,4-, Ifnγ^−/−^ deficient and wild-type mice following 18 Gy whole thorax irradiation		In response to radiation, irradiated IL-17^−/−^ mice showed both decreased lung fibrosis and pneumonitis	IL-17A was a key promoter for RIPI
Zhang et al.,^70^ 2023	A genome-wide microarray analyses in RIPI mice induced by whole thoracic irradiation with a single fraction of 20 Gy X-ray		The IL-17A signaling pathway was upregulated	The IL-17A signaling pathway was activated in RIPI
Liu et al.,^69^ 2024	PD-1 KO (PD-1^−/−^) and wild-type mice exposure to 8 Gy × 3 doses in both lungs		PD-1^−/−^ mice exhibited a high rate of RIPI following irradiation treatment and a significantly increased expression level of IL-17A. Administration of anti-IL-17A antibody decreased RIPI-related factors and prolonged the survival of mice	IL-17A was a key promoter for RIPI
Liu et al.,^38^ 2025	C57BL/6J mice and human lung epithelial cells (BEAS-2B) were exposed to irradiation with or without recombinant IL-17A	Autophagy markers were measured by diverse techniques/methods	High expression of IL-17A and low levels of autophagy were identified in the lung tissues of mice with acute RIPI. IL-17A suppressed autophagic activity by inhibiting the PP2A B56α-mTOR pathway in BEAS-2B cells exposed to irradiation	The promoting effect of IL-17 on RIPI is associated with a low level of autophagy in the process of RIPI
RIII				
Bessout et al.,^53^ 2015	Severe colonic mucosal injury and fibrosis were induced in Sprague–Dawley rats and nude rats treated with radiation		Th 17 cells in radiation-induced bowel tissues were significantly activated and increased in rats. In vitro studies showed that IL-17A can directly act on colonic smooth muscle cells to induce expression of proinflammatory genes that could participate in the development of RIII. After Ad-MSCs treatment, the Th17 population and IL-17A level were reduced, and the histological injury of RIII was attenuated	The activation of Th17 cells and IL-17A were associated with the process of RIII in rats

Ad-MSCs, adipose tissue mesenchymal stromal cells; HMGB1, high mobility group box-1; IL, interleukin; IMRT, intensity modulated RT; NSCLC, non-small cell lung cancer; PD-1, programmed cell death protein 1; RID, radiation-induced dermatitis; RIEI, radiation-induced esophageal injury; RIII, radiation-induced intestinal injury; RIPI, radiation-induced pulmonary injury; RITIs: radiation-induced tissue injuries; RT, radiotherapy; SBRT, stereotactic body RT.

### IL-17A protects against RITIs in the oral and intestinal mucosa

#### Radiation-induced oral mucositis

Radiation-induced oral mucositis (RIOM) is a common RITI that occurs in the normal oral mucosa, tongue, and pharynx in patients with certain types of cancers receiving RT. Accumulating evidence has revealed that RIOM starts with an acute inflammatory process after irradiation, with high populations of immune cells and high levels of proinflammatory mediators, including cytokines and chemokines, in the normal oral tissues.^76,77^

Recent studies have revealed that the involvement of IL-17A in the pathogenesis of RIOM. Saul-McBeth et al.^44^ reported that mice deficient in IL-17 receptor A (IL-17RA KO) exhibited more severe pathological features of RIOM in response to head and neck irradiation through multiple mechanisms, including restoration of damaged epithelia and control of the neutrophil response. They confirmed that oral mucositis after irradiation in IL-17RA KO mice was associated with a higher density of neutrophils and more severe immunopathological features in oral tissues than in WT mice.^44^ In addition, while radiation enhanced the expression of the IL-17-related transcriptional genes during the process of RIOM, the lack of IL-17RA in these mice increased the IL-1-mediated recruitment of neutrophils in oral tissues.^44^ These findings support the protective role of IL-17A against RIOM development in mice.

#### Radiation-induced intestinal injury

Radiation-induced intestinal injury (RIII) is a common intestinal complication frequently observed in patients undergoing pelvic and abdominal RT.^78^ The effects of Th17 cells and IL-17A on RIII have been evaluated and are inconsistent.^45,53^ Bessout et al.^53^ reported that an increased population of infiltrating Th17 cells and IL-17A levels in intestinal tissues were closely associated with an impaired tissue regeneration capacity and development of fibrosis in the rat intestine after irradiation, suggesting that IL-17A promotes RIII. In a review article, Shao et al.^79^ also speculated that the contribution of radiation-induced bacterial dysbiosis to RIII might be through the enhanced accumulation of Th17 cells and increased production of IL-17A in the intestine. In contrast, Kempen et al.^80^ recently showed that IL-17A signaling is important for maintaining intestinal barrier integrity in Paneth cell-specific IL-17RA KO mice after gamma radiation-induced injury. More recently, Huang et al.^45^ showed that Scleroglucan^81^ protects against RIII through the upregulation of Th17 cells and IL-17A expression, as well as the activation of NF-κB signaling pathways in the gut tissues of mice. Intraperitoneal injection of anti-mouse IL-17A antibody reverses this protective effect of Scleroglucan.^45^ Based on the results of the current study, Th17 cells and their main cytokine product, IL-17A, were most likely to play a protective role in RIII. However, evidence supporting this hypothesis requires further experimental studies.

#### RITIs in other tissues

IL-17A activation reduces the impairment caused by radiation and enhances the recovery of hematopoietic function after gamma irradiation,^82^ suggesting IL-17A is a protective factor for hematopoietic tissue after RT. Because RT is toxic and causes tissue injury, identifying the effect of IL-17A in different tissues may help researchers and clinicians understand the immunopathogenesis of RITI in patients with cancer after RT.

Table 2 summarizes the studies that describe a protective effect of IL-17A on certain types of RITIs.

**Table 2.** Main studies on IL-17A are known as a protective factor against the process of RITIs.

Study (Ref.) and year	Study models		Main findings	Conclusion
	Animal	Human		
RIOM				
Saul-McBeth et al.,^44^ 2021	RIOM was induced in IL-17 receptor A-deficient (IL-17RA KO) mice by exposure to head and neck irradiation (22.5 Gy at the rate of 1000 cGy/min in a single fraction)		Elevated level of IL-17A expression in response to head and neck irradiation. RIOM was reduced in IL-17RA KO mice. Severity of RIOM in IL-17RA KO mice was higher than that in wild-type mice, accompanied by increased recruitment of neutrophils derived from IL-1R	IL-17A was a protective factor for RIOM
RIPI				
Xiong et al.,^74^ 2015	Treg depletion in mice by intraperitoneal injection with anti-CD25 monoclonal antibody 2 h after 20 Gy 60CO γ-ray thoracic irradiation		Depletion of Tregs delayed the process of radiation-induced pulmonary fibrosis by increasing infiltration of CD4^+^ Th and Th17 cells in the lung	Th17 cells were the anti-fibrotic factors and protective factors for RIPI
RIII				
Kempen et al.,^80^ 2022	Entire gut epithelium (IL-17ra^fl/fl^; villin-cre) and Paneth cell-specific IL-17RA KO mice (IL-17RA^fl/fl^; Defa6-cre^+^) after gamma radiation-induced injury		Increased microbial dissemination to liver and spleen in these mice compared to their respective control mice. Reduced expression of Lyz1 3 days after radiation in the terminal ileum of IL-17RA KO mice suggested impaired Paneth cell function	IL-17A was a protective factor in Paneth cell function that contributes to the maintenance of intestinal mucosa integrity
Huang et al.,^45^ 2024	Mice were randomly grouped and intraperitoneally injected with phosphate-buffered saline buffer, Scleroglucan or anti-mouse IL-17A antibody (10 mg/kg) after irradiation (10.0 Gy)		Increased number of Th17 cells and elevated level of IL-17A contributed to the protective effect of Scleroglucan on RIII. Such a protective effect could be reversed by blocking the IL-17A signal	IL-17A was a protective factor for the process of RIII
Tan et al.,^82^ 2006	IL-17R knockout (IL-17Ra^−/−^) mice challenged with gamma irradiation		Hemopoietic toxicity is significantly more pronounced in IL-17Ra^−/−^ mice as compared with control mice. Similar results were also observed in wild-type mice after in vivo blockade of IL-17R	IL-17A is a protective factor that can help the recovery of hemopoietic function after gamma irradiation

IL, interleukin; RIII, radiation-induced intestinal injury; RIOM, radiation-induced oral mucositis; RIPI, radiation-induced pulmonary injury; Tregs, regulatory T-cells.

Overall, the current literature suggests the involvement of IL-17A in RITI immunopathogenesis, which can be both promotive and protective in different tissues, or even in the same type of tissue. These conflicting results obtained in different tissues may reflect the heterogeneity in the sensitivity to irradiation.^37,83^

## Proposed mechanisms for IL-17A in the pathogenesis of RITIs

### Proposed mechanisms for the promotive effect of IL-17A on RITIs in normal skin, esophagus, and lung tissues

Radiation results in significant activation of IL-17A transcription signal in different types of immune cells, for example, γδ-T cells and Th17 cells, increasing the expression of IL-17A.^43,47,61,65^ Studies have shown that after irradiation, the activated IL-17A signal triggers an inflammatory cascade, initiating the process of inflammation in normal tissues.^40,65,68^ Excessive and prolonged inflammatory responses may cause cell death and functional defects,^37,38^ and the proinflammatory cytokines released by the dead cells further amplify tissue inflammation and cell damage.^84^ Furthermore, RT-induced DNA damages, such as double-strand breaks, cross-linking, and base impairment, lead to increased mutations and cell cycle arrest, thereby hindering cell division and causing cell death in the surrounding normal tissues.^85^ Studies have also shown that radiation can significantly activate both intracellular and extracellular levels of free radicals, such as ROS, reactive nitrogen species, and nitric oxide (NO),^37,85,86^ in addition to directly causing DNA damage, triggering cellular oxidative stress and inflammatory responses.^87,88^ Furthermore, radiation-induced free radicals can damage the cell membrane and induce cell death in both normal tissues and immune cells via necrosis and programmed cell death. If radiation-induced DNA injury is not repaired promptly and appropriately, the cell membrane will lose integrity and eventually break down, allowing high amounts of proinflammatory cytokines, such as IL-17A, out of dead cells.^83^ Interestingly, radiation-induced free radicals and IL-17A can stimulate the production of each other and activate relevant signaling pathways to trigger inflammatory cascades and initiate inflammation in normal tissues.^89^ Moreover, studies have shown that IL-17A can modulate the differentiation and activation of stromal fibroblasts to promote lung fibrosis,^68,90^ which contributes significantly to the pathogenesis of radiation-induced fibrosis in normal lung tissues.^37^ Finally, recent studies have confirmed that radiation greatly affects the composition of the gut microbiota and induces dysbiosis,^91^ Th17-favored bacteria promote the generation of Th17 cells that produce IL-17A, leading to increased levels of IL-17A in the small intestine tissues.^92,93^ Therefore, it is proposed that gut bacterial dysbiosis induced by either total body or abdominal irradiation may cause accumulation of IL-17A-producing Th17 cells and radiation-induced enteritis.^80^ Furthermore, the importance of microbial-IL-17A crosstalk has recently received particular attention in chemotherapy-induced lung injury. Yang et al.^94^ showed that dysregulated lung commensal bacteria upregulated the expression of IL-17A and IL-17B in macrophages residing in the lung tissues, which is associated with bleomycin-induced lung inflammation and injury by enhancing neutrophil recruitment. These findings highlight the need for considering and evaluating the role of commensal bacterial profile changes in response to RT in the process of RITIs.

**Figure 1.**
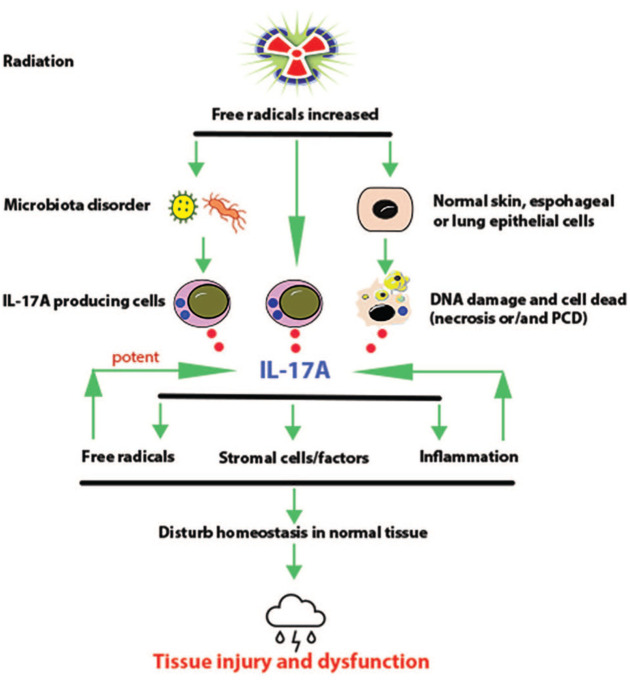
Schematic representation of proposed mechanisms for the promotive effect of IL-17A on the pathogenesis of RITIs in the skin, esophageal, and lung tissues. Overproduction of free radical substances induced by ionizing radiation can directly stimulate the IL-17A-producing cells to produce IL-17A and indirectly induce cell death in the normal tissues via both necrosis and PCD, which allows high amounts of IL-17A out of dead cells. Interestingly, radiation-induced free radicals and IL-17A can stimulate each other and activate relevant signaling pathways to trigger inflammatory cascades and initiate inflammation in normal tissues. In addition, free radical substances can cause microbiota disorder in the gut and normal tissues and activate signaling pathway of IL-17A synthesis in IL-17A-producing cells. Moreover, IL-17A can activate stromal myofibroblasts for the process of fibrosis in normal lung tissues. Therefore, it is proposed that multiple mechanisms are involved in the pathogenesis of RITIs in the skin, esophageal, and lung tissues. IL, interleukin; PCD, programmed cell death; RITIs, radiation-induced tissue injuries.

Figure 1 presents a summary of the proposed mechanisms that explain the promotive effects of IL-17A on the pathogenesis of RITIs in skin, esophageal, and intestinal tissues.

### Proposed mechanisms for the protective effects of IL-17A against RITIs in normal oral and intestinal mucosa

As described earlier, irradiation-induced activation of IL-17A has been shown to protect against RIOM and RIII development, although the exact mechanisms remain known.^44,45,95^ The proposed mechanisms include: the ability of IL-17A to stimulate the proliferation of oral epithelial cells and maintain mucosal immune homeostasis, which may contribute to the restoration of damaged oral mucosal cells.^44^ In the intestine, IL-17A helps maintain the integrity of the intestinal mucosal barrier^95^ and thereby activates intestinal immune function.^45^ It has previously been identified that tissue stem cells play a key role in maintaining tissue homeostasis by generating novel cells to replace the damaged cells.^96,97^ Recent studies have demonstrated that radiation can significantly affect stem cell functions and the repair of RITIs.^98^ In a study on the organoid formation capacity of irradiated primary bronchial epithelial cells cultured at the air-liquid interface, Kuipers et al.^99^ have demonstrated that ionizing radiation significantly harms the function of resident bronchial epithelial stem cells, thereby affecting the repair of radiation-induced cell injury in lung tissues. IL-17A may regulate the differentiation, expansion, and mobilization of stem cells^100^ to promote the repair of acutely and chronically damaged cells in response to various dangerous factors.^101,102^ In addition, IL-17A modulates the activity of mesenchymal stem cells (MSCs), a heterogeneous population of stromal stem cells with different multi-potential properties. MSCs play an essential role in tissue repair^103^ by directly replacing damaged cells and/or indirectly modulating the differentiation and function of stromal cells,^104^ through the secretion of anti-inflammatory and anti-fibrotic factors.^103^ IL-17RA is highly expressed in stromal cells, including MSCs^100^ and mediates the IL-17A-driven proliferation of MSCs.^105^ Ma et al.^106^ reported that IL-17A can improve the migration and homing ability of MSCs, indicating a possible regulatory effect of IL-17A on MSCs, which may contribute to their protective effects against RITIs in normal tissues such as the oral and intestinal mucosa. Therefore, evaluation of the relevant effects and mechanisms of MSCs modulated by IL-17A in the protection against RITIs in the normal oral and intestinal mucosa should be prioritized in the future. More recently, Konieczny et al.^107^ reported that IL-17A produced by skin-resident γδ-T cells aids in the adaptation of skin epithelium to hypoxia and repair of skin tissue through the activation of hypoxia-inducible transcription factor 1α (HIF1α) in the wound-edge epithelium, which depends on the IL-17RC. These findings provide further evidence supporting the importance of IL-17A in skin tissue repair. It would be interesting to evaluate the role of the IL-17A–HIF1α axis in other RITIs.

**Figure 2.**
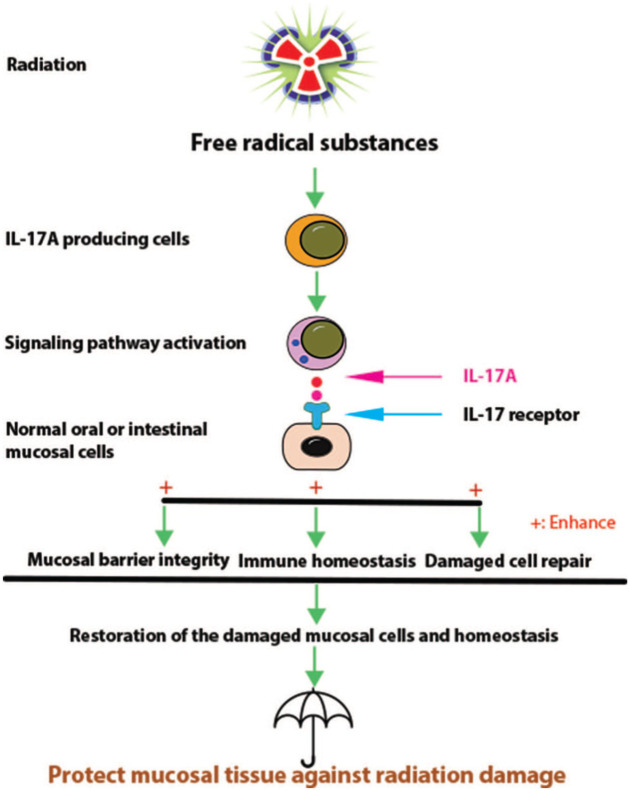
Schematic representation of proposed mechanisms for the protective effect of IL-17A on the pathogenesis of RITIs in the oral and intestinal mucosa. Radiation-induced free radical substances cause overproduction of IL-17A, which protects tissues against RITIs by maintaining mucosal barrier integrity, keeping immune balance, and enhancing damaged cell repair through the IL-17 receptors expressed on targeted cells. These effects contribute to the maintenance of tissue homeostasis and ultimately reduce the development of RITIs in the oral and intestinal mucosa after RT. IL, interleukin; RITIs, radiation-induced tissue injuries; RT, radiotherapy.

Figure 2 presents a schematic summary of the proposed mechanisms underlying the protective effects of IL-17A on RITIs in normal oral and intestinal mucosa.

## Translational significance and perspectives of targeting the IL-17A signal in RITIs

### Can IL-17A be a predictive biomarker for the development of RITIs?

The prognostic value of IL-17A as a biomarker has been intensively evaluated in various types of cancers, and most studies have suggested that changes in IL-17A levels are associated with certain clinical parameters, such as angiogenesis status, disease stage, survival time, and immune function in patients with cancer.^108–111^ In addition, IL-17A levels can be used for predicting clinical response to anticancer therapies.^108,112^ More recently, Yu et al.^112^ reported that elevated IL-17A levels predict aggressive tumor behavior and resistance to neoadjuvant therapy in patients with breast cancer.

Fessé et al.^113^ recently reviewed and summarized the current findings of the association between the levels of inflammatory biomarkers, including cytokines, and the risk of RITIs in cancer patients with RT. Their analysis suggested that RT-induced inflammatory biomarker expression might predict both early and late toxicity outcomes in patients with cancer undergoing RT. Therefore, there is great interest in evaluating the potential of IL-17A-producing cells and their levels as biomarkers for predicting RT toxicity and RITI development.^37,43,44,53–55^ Mieczkowska et al.^40^ confirmed that increased levels of IL-17A positively correlated with RID severity in irradiated skin, highlighting its potential as a predictive biomarker of RID development/progression. Wang et al.^55^ revealed that the ratio of Th17/Tregs in the blood of patients with esophageal cancer could predict the risk of developing radiation-induced pneumonia after RT. However, large cohort clinical studies supporting the use of IL-17A as a biomarker for RITIs are lacking. Therefore, longitudinal studies to validate the value of IL-17A as a biomarker of RT-induced tissue inflammation and RITIs are needed, which will ultimately improve the therapeutic response and clinical outcomes in patients with cancer undergoing RT.

In addition, IL-17A and other proinflammatory cytokines with overlapping biological functions might form a complex network that participates in the regulation of RITIs development^114^ making the evaluation of IL-17A’s predictive value in RITIs challenging.

### Can IL-17A/IL-17R activation be a therapeutic candidate for RITIs?

Proinflammatory cytokines have been proposed as potential targets for the management of RITIs.^115^ Clinically, neutralizing antibodies that can block IL-17, IL-17RA, and/or IL-17RC signals have been widely used and shown a reduced severity and improved remission in inflammatory diseases such as inflammatory bowel diseases, psoriatic arthritis, rheumatoid arthritis, and systemic lupus erythematosus.^30,116^

The effects of blocking IL-17A and IL-17 receptor signals on severity and the development of certain types of RITIs have been evaluated in preclinical models. Animal studies have shown that mice receiving IL-17A-neutralizing antibody injection, as well as mice genetically knocking out IL-17 receptor C (IL-17RC KO), resulted in a significantly less severe RID after irradiation.^40^ Furthermore, Wang et al.^68^ found that neutralizing the IL-17A signaling by the administration of anti-IL-17A antibodies before exposure to irradiation resulted in a significant reduction in the percentage of grade II and III alveolitis in irradiated mice compared to either the RT alone or placebo groups. Also, pretreatment with anti-IL-17A antibodies significantly decreased the mortality rate within 6 months in mice with radiation-induced pneumonitis.^68^ Wang et al.^67^ further showed that the protective effect of blocking HMGB1 signaling, an important nuclear protein involved in the process of lung tissue injury, occurred through the reduced expression of IL-17A in a similar RIPI murine model. Similar findings were also reported in RID mice. Pirtle et al.^117^ found that mice with an IL-17RC genetic deficiency and mice receiving IL-17A-neutralizing antibodies exhibited a significantly reduced rate of RID. These findings suggest that blocking IL-17A/IL-17R signaling may prevent or reduce the severity of RT-induced skin tissue injury. Therefore, it would be interesting to precisely examine the therapeutic efficacy of blocking IL-17A/IL-17R signaling in RITIs in the future.

More recently, Khalil et al.^118^ reported that gamma irradiation-induced increased levels of IL-17A in irradiated Wistar rats, which were decreased by pretreatment with resveratrol at concentrations of 10 and 100 mg/kg. Therefore, it would be interesting to evaluate the therapeutic effects of resveratrol on RITIs in preclinical animal models. Furthermore, primary studies have revealed that crosstalk between the gut microbiota and IL-17A is involved in the pathogenesis of certain types of RITIs, such as radiation-induced enteritis.^80^ These findings may provide the possibility of modulating the expression of IL-17A by fecal microbiota transplantation to reprofile the composition of the gut microbiota, thereby reducing the development of RITIs. In addition, the therapeutic efficacy of targeting IL-17A/IL-17 receptor signaling together with MSCs therapy to inhibit RITIs also remains to be evaluated. Finally, several studies have reported the protective role of IL-17A in the pathogenesis of RIOM and RIII.^44,45,95^ Therefore, evaluation of the therapeutic efficacy of enhancing the IL-17A signaling pathway in treating other RITIs, especially RIOM, in preclinical animals is necessary in the future.

However, there are some challenges associated with these approaches. First, the therapeutic efficacy of blocking IL-17A/IL-17 receptor signals has only been observed in a few types of RITIs, such as RIPI and RID, in animal models.^37^ Whether other types of RITIs can benefit from such immunotherapy remains unclear. Thus, there is a clear need for further studies to elucidate the exact role and effect of IL-17A in other types of RITIs. Second, IL-17A, together with other proinflammatory cytokines, forms a complex network to modulate the process of radiation-induced inflammation and injury in normal tissues. Targeting the IL-17A signaling pathway alone may only attenuate but not completely block the pathological process of RITIs. Thus, evaluation of the synergistic efficacy of combinational strategies in RITIs provides key information for the design of novel immunotherapeutic strategies.

## Conclusion

Recent scientific findings have provided emerging evidence to suggest that IL-17A activation by ionizing radiation plays a complex dual role in different RITIs, exhibiting both promotive and protective functions. Most supportive evidence for its promotive function was observed in preclinical RIPI and RID animal models, suggesting a possible beneficial effect from blocking IL-17A or IL-17 receptor signals. However, the protective function of IL-17A is also observed in other types of RITIs, such as RIOM and RIII. These findings suggest that this dual nature is tissue-dependent. Therefore, future studies that focus on dissecting the dual roles of IL-17 in different RITIs may help to develop personalized therapeutic options by targeting IL-17A/IL-17 receptor signaling pathway for better management of different RITIs.

## Declarations

**Ethics approval and consent to participate:** Not applicable.

**Consent for publication:** Not applicable.

**Availability of data and materials:** Not applicable.
